# Effects of Response to 2014–2015 Ebola Outbreak on Deaths from Malaria, HIV/AIDS, and Tuberculosis, West Africa

**DOI:** 10.3201/eid2203.150977

**Published:** 2016-03

**Authors:** Alyssa S. Parpia, Martial L. Ndeffo-Mbah, Natasha S. Wenzel, Alison P. Galvani

**Affiliations:** Yale School of Public Health, New Haven, Connecticut, USA

**Keywords:** Ebola virus, malaria, HIV/AIDS and other retroviruses, tuberculosis and other mycobacteria, healthcare, mathematical model, mortality rate, viruses, parasites, parasitic, vector-borne infections, mycobacteria, zoonoses

## Abstract

Response to the 2014–2015 Ebola outbreak in West Africa overwhelmed the healthcare systems of Guinea, Liberia, and Sierra Leone, reducing access to health services for diagnosis and treatment for the major diseases that are endemic to the region: malaria, HIV/AIDS, and tuberculosis. To estimate the repercussions of the Ebola outbreak on the populations at risk for these diseases, we developed computational models for disease transmission and infection progression. We estimated that a 50% reduction in access to healthcare services during the Ebola outbreak exacerbated malaria, HIV/AIDS, and tuberculosis mortality rates by additional death counts of 6,269 (2,564–12,407) in Guinea; 1,535 (522–2,8780) in Liberia; and 2,819 (844–4,844) in Sierra Leone. The 2014–2015 Ebola outbreak was catastrophic in these countries, and its indirect impact of increasing the mortality rates of other diseases was also substantial.

## Abstract 2

Reduced access to healthcare during the outbreak substantially increased mortality rates from other diseases.

The 2014–2015 Ebola outbreak in West Africa debilitated the healthcare systems of affected countries, hampering diagnosis and treatment for endemic diseases such as malaria, HIV/AIDS, and tuberculosis (TB) (*1*,*2*). The deaths of several healthcare workers early in 2014, as well as the strain on healthcare facilities caused by increased numbers of patients and decreased staff, resulted in the closure of many clinics and the interruption of routine health delivery services, including HIV testing, childhood vaccinations, and maternity care. Fear of Ebola transmission decreased outpatient attendance to as low as 10% (*3*,*4*). Surveys conducted by the United Nations in Guinea, Liberia, and Sierra Leone found a substantial decline in the number of persons seeking healthcare because they feared nosocomial Ebola transmission (*1*). In addition, mandatory curfews, border closures, and disruption of transportation routes made obtaining medical services or continuing drug therapy challenging. The reduced demand for and availability of healthcare in the Ebola-affected regions exacerbated the severity of illness and number of deaths caused by malaria, HIV/AIDS, and TB.

Malaria, HIV/AIDS, and TB are 3 of the most prevalent infectious diseases in West Africa (5). Halting the spread of these pathogens is a 2015 Millennium Development Goal and the priority of the Global Fund (*5*). Among children <5 years of age, the annual prevalence of malaria is estimated to be 44% in Guinea, 45% in Liberia, and 43% in Sierra Leone (*6*–*8*). HIV/AIDS prevalence among persons 15–49 years of age is 1.7% in Guinea, 1.1% in Liberia, and 1.6% in Sierra Leone (*9*,*10*), and TB prevalence across all ages in the 3 countries is 0.24%, 0.44%, and 0.43%, respectively (*11*). Without treatment, annual mortality rates are reported to be as high as 80% for severe malaria, 25% for HIV/AIDS, and 40% for TB (*12*–*14*). In addition, HIV/AIDS and TB require long-term treatment, while malaria reinfections require repeated treatment. Thus, disruptions of healthcare services that interrupt treatment may substantially increase the number of deaths associated with malaria, HIV/AIDS, and TB.

To estimate the indirect health burden of the 2014–2015 Ebola outbreak in Guinea, Liberia, and Sierra Leone, we developed computational simulation models for malaria, HIV/AIDS, and TB. We used epidemiologic data obtained from the most recent reports of the World Health Organization (WHO) Demographic Health Surveys (DHS) (Table 1; Technical Appendix Table 1) (*15*–*23*) and the Global Burden of Disease study (*5*,*24*) on disease prevalence, incidence, and mortality rates to calibrate our models to the disease burden before the Ebola outbreak. We then projected the calibrated models to estimate the effect that the Ebola outbreak had on disease-related deaths through reduced access to treatment for varying reductions in treatment coverage.

**Table 1:** Parameter estimates and distributions for models of malaria in Guinea, Liberia, and Sierra Leone, measuring impact of response to the 2014–2015 Ebola outbreak on deaths*

Malaria-related parameter estimates	Value	Reference
Probability of death without treatment, range		
Uncomplicated malaria	0.005–0.02	(*15*)
Severe malaria	0.45–0.80	(*13*)
Probability of death while undergoing treatment, range		
Uncomplicated malaria	0.00024–0.00112	(*16*)
Severe malaria	0.05–0.2	(*17*)
Probability of progressing from uncomplicated to severe malaria given no treatment	0.03–0.13	(*13**,**18*)
Proportion of case-patients with fever attributable to Malaria	0.01–0.11	(*18**,**19*)
Probability of spontaneous recovery from uncomplicated malaria	0.10–0.20	(*18*)
Probability of treatment for severe malaria	0.60–0.80	(*20*)
Age-specific probabilities†		
Guinea		
Development of fever within 2 weeks (β distribution)		(*21*)
<1 y	376/1,453	
1–2 y	476/1,296	
2–3 y	406/1,192	
3–4 y	337/1,253	
4–5 y	301/1,252	
Receiving treatment for malaria before Ebola outbreak		
<1 y	0.128–0.221	(*21*)
1–2 y	0.194–0.334	
2–3 y	0.159–0.260	
3–4 y	0.198–0.309	
4–5 y	0.163–0.271	
Liberia		
Development of fever within 2 weeks (β distribution)		(*22*)
<1 y	391/1,333	
1–2 y	429/1,272	
2–3 y	309/1,085	
3–4 y	327/1,198	
4–5 y	273/1,159	
Receiving treatment for malaria before Ebola outbreak		
<1 y	0.296–0.381	(*22*)
1–2 y	0.461–0.603	
2–3 y	0.393–0.538	
3–4 y	0.449–0.618	
4–5 y	0.521–0.624	
Sierra Leone		
Development of fever within 2 weeks (β distribution)		(*23*)
<1 y	576/2,406	
1–2 y	706/2,169	
2–3 y	570/2,011	
3–4 y	493/2,237	
4–5 y	406/1,991	
Receiving treatment for malaria before Ebola outbreak		(*23*)
<1 y	0.301–0.395	
1–2 y	0.376–0.502	
2–3 y	0.354–0.484	
3–4 y	0.395–0.543	
4–5 y	0.376–0.501	

*See Technical Appendix Table 1 for HIV/AIDS and tuberculosis parameter estimates and distributions. †For fever, values are no. persons in that age group that had a fever 2 weeks before the survey/total no. persons in age group.

## Methods

We developed 3 computational simulation models from a population perspective of disease burden: a disease progression model for malaria and 2 decision tree models for HIV/AIDS and active TB cases. In all models, the decision node branches represent temporal intervals before and during the Ebola outbreak. For each disease, we conservatively considered deaths only for the highest risk groups: malaria among children >5 years of age, HIV/AIDS among persons 15–49 years of age, and active TB in all age groups.

We used these models to estimate the number of deaths from malaria, HIV/AIDS, and TB that would have occurred in Guinea, Liberia, and Sierra Leone from March 2014, the start of the Ebola outbreak, through March 2015. We compared this estimate with a scenario of reduced access to health services from June 2014, when Ebola was reported in the major cities of the 3 countries, through March 2015, when the outbreak was tapering (*1*,*25*). In other settings, this modeling approach has been used to assess the burden of illness for malaria (*18*), TB (*26*), and HIV/AIDS (*27*).

### Malaria

**Figure 1:**
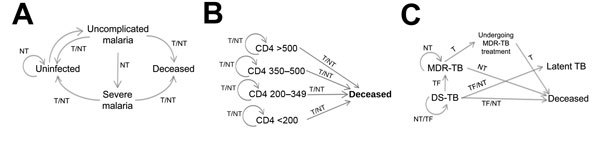
Health state transition diagrams for A) malaria, B) HIV/AIDS, and C) tuberculosis models for disease transmission and progression. T, patient was treated; NT, no treatment was provided; TF, treatment failure or default.

To evaluate the death rate of malaria, we developed a model (Figure 1, panel A) that projects the impact of malaria among children <5 years of age with an 8-day time step using a Markov model. The 8-day time step corresponds to the average duration of a malaria episode among children (*28*). Our model tracks 4 health states: uninfected, infected with uncomplicated symptomatic malaria, infected with severe malaria, and deceased. Based on time-dependent probabilities of infection, treatment, or disease progression, persons may transition through these health states over the course of a specified time horizon. We used an age-specific infection probability to account for naturally acquired immunity (*29*). Upon infection, children transition to the health state of uncomplicated malaria, from which they can recover, progress to severe malaria, or die from their infection. Similarly, children with severe malaria may either recover or die. For simplicity, we assumed that severe malaria can only occur as a progressive state from uncomplicated malaria. Probabilities of recovery and death depend on whether malaria is uncomplicated or severe and on whether or not children receive treatment.

In sub-Saharan Africa, most cases of uncomplicated malaria are treated presumptively on the basis of symptoms rather than a positive test or formal medical diagnosis, and treatment medication is purchased from a local store or pharmacy (*30*). Because of this, we conservatively assumed that disruption of health services from the Ebola outbreak only affected treatment for severe malaria.

### HIV/AIDS

We developed a decision tree model to estimate the impact of reduced antiretroviral treatment (ART) on HIV/AIDS-related deaths in Guinea, Liberia, and Sierra Leone (Figure 1, panel B). We measured the HIV/AIDS mortality rates in terms of related deaths among persons 15–49 years of age with HIV/AIDS. We took into account both the distribution of CD4 count (>500, 350–500, 200–349, <200 cells/mm^3^), and probabilities dependent on CD4 counts of dying without ART, ART failure, and dying while receiving ART (Technical Appendix Table 1). Following WHO recommendations for ART initiation that were in place during the pertinent time period (2013–2015), we assumed that persons did not initiate ART unless their CD4 count was <500 cells/mm^3^ (*31*). We did not incorporate HIV progression because the progression rate is slow compared with the time frame of interest (*32*).

### Tuberculosis

We developed a decision tree model to estimate the impact of reduced treatment coverage on the TB burdens in Guinea, Liberia, and Sierra Leone throughout the Ebola outbreak (Figure 1, panel C). We focused on persons with active TB at the start of the Ebola outbreak. We distinguished between infection with drug-susceptible TB and infection with multidrug-resistant TB (MDR TB). We assumed that without treatment, persons infected with DS-TB would either recover naturally, remain infected with drug-susceptible TB, or die at rates based on results of clinical studies. MDR TB case-patients were assumed to either remain infected or die from their infection over the course of the Ebola outbreak.

We assumed that persons with drug-susceptible TB who receive treatment can either be successfully treated, be unsuccessfully treated and remain alive, or die. Those who experience treatment failure or discontinue treatment before taking the full course may progress to MDR TB, and then may seek treatment for MDR TB. We assumed that persons who were infected with MDR TB at the start of the Ebola outbreak would remain infected or die during the 9-month time horizon of our analysis if they were not treated. Given that MDR TB requires 24 months of treatment (*33*), we assumed that persons who received treatment would remain under treatment throughout the 9 months regardless of whether they were still infected or had regressed to latent TB.

### Model Calibration

We parameterized the model with a distribution of values for the epidemiologic parameters and population-at-risk estimates from published literature (Table 1; Technical Appendix Tables 1, 2). We calibrated our models by comparing annual deaths before the Ebola outbreak with estimates from the 2013 Global Burden of Disease studies for Guinea, Liberia, and Sierra Leone (*5*,*24*). Each input parameter distribution was sampled 5,000 times. The samples for which the death counts fell within the 95% CI of the Global Burden of Disease studies were selected for our uncertainty analysis (Table 2). Model iterations simulated the disease and treatment events of 5,000 persons for all diseases, resulting in a total of 25 million realizations. The incidence of malaria episodes in our model was 0.3 (95% CI 0.1–0.6) episode/person-year in Guinea, 0.3 (95% CI 0.1–0.6) episode/person-year in Liberia, and 0.3 (95% CI 0.2–0.5) episode/person-year in Sierra Leone, consistent with empirical estimates from West Africa (*29*). On the basis of reports regarding the reduction of routine healthcare services and access to treatment in Guinea, Liberia, and Sierra Leone during the Ebola outbreak (*1*), we considered a 50% reduction for malaria, HIV/AIDS, and TB treatment in our base case analysis.

**Table 2:** Model calibration results compared to empirical data from the 2013 Global Burden of Diseases Study of situation before the start of the 2014–2015 Ebola outbreak, for deaths due to malaria, HIV/AIDS, and tuberculosis*

Country	Average no. deaths (range)							
	Malaria			HIV/AIDS			Tuberculosis	
	GBD Study	Model		GBD Study	Model		GBD Study	Model
Guinea	11,591 (4,817–19,932)	15,200 (4,370–20,330)		4,913 (2,774–7,956)	5,832 (2,916–7,920)		3,479 (2,696–4,378)	3,519 (2,698–4,382)
Liberia	2,111 (603–4,420)	2,100 (700–4,200)		1,741 (1,062–2,652)	1,548 (1,062–2,652)		1,394 (1,081–1,843)	1,400 (1,076–1,850)
Sierra Leone	7,011 (2,591–12,613)	5,400 (2,430–12,600)		3,419 (1,830–5,494)	3,132 (1,836–5,472)		1,986 (1,522–2,579)	1,978 (1,514–2,575)
*GBD, Global Burden of Diseases (*5*,*24*).								

### Multivariate Sensitivity Analysis

To evaluate the effect of each epidemiologic parameter on the relative excess deaths from Ebola, we conducted a multivariate sensitivity analysis using partial rank correlation coefficient (PRCC). PRCC measures the strength of monotonic association between the input parameters and output variable (*28*). The larger the PRCC, the stronger the influence of the model parameter on mortality rates from the disease of interest: malaria, HIV/AIDS, or TB.

## Results

### Malaria

We estimated that a 50% reduction in treatment coverage during the Ebola outbreak would lead to the deaths of 12,825 (95% CI 4,845–21,945) children <5 years of age from malaria in Guinea, 2,573 (95% CI 735–5,040) in Liberia, and 4,860 (95% CI 2,700–9,450) in Sierra Leone. We estimated that malaria-attributable mortality rates increased by 48.0% (95% CI 4.9%–93.8%) in Guinea, 53.6% (95% CI 4.8%–145.5%) in Liberia, and 50.0% (95% CI 5.0%–118.8%) in Sierra Leone. This increase represents 4,275 (95% CI 570–9,405) additional deaths in Guinea, 788 (95% CI 105–1,890) in Liberia, and 1,755 (95% CI 135–2,970) in Sierra Leone (Table 3).

**Table 3:** Deaths from malaria, HIV/AIDS, and tuberculosis correlated with a 50% reduction in treatment coverage attributable to response to the Ebola outbreak, West Africa, 2014–2015

Country and disease	Total no. estimated deaths	No. deaths (95% CI) attributable to outbreak	% Change in attributable deaths (95% CI)	Total deaths attributable to outbreak
Guinea				6,269 (2,564–12,407)
Malaria	12,825 (4,845–21,945)	4,275 (570–9,405)	48.0 (4.9–93.8)	
HIV/AIDS	5,151 (3,099–7,333)	713 (58–1,528)	16.2 (1.3–30.2)	
Tuberculosis	3,463 (2,808–4,349)	1,281 (877–1474)	51.1 (44.7–70.5)	
Liberia				1,535 (522–2,878)
Malaria	2,573 (735–5,040)	788 (105–1,890)	53.6 (4.8–145.5)	
HIV/AIDS	1,198 (851–1,841)	155 (23–297)	13.0 (2.6–25.4)	
Tuberculosis	1,553 (1,216–1,875)	592 (394–691)	59.0 (47.9–77.4)	
Sierra Leone				2,819 (844–4,844)
Malaria	4,860 (2,700–9,450)	1,755 (135–2970)	50.0 (5.0–118.8)	
HIV/AIDS	2,621 (1,390–4,183)	223 (29–504)	9.1 (1.6–19.1)	
Tuberculosis	2,164 (1,815–2,548)	841 (680–1,010)	61.4 (49.2–87.6)	

### HIV/AIDS

Given a 50% reduction in ART coverage during the Ebola outbreak, we estimated that 5,151 (95% CI 3,099–7,333) adults 15–49 of age would have died in Guinea, 1,198 (95% CI 851–1,841) in Liberia, and 2,621 (95% CI 1,390–4,183) in Sierra Leone. The increase in HIV/AIDS deaths attributable to this reduction in ART coverage was estimated to be 16.2% (95% CI 1.3%–30.2%) in Guinea, 13.0% (95% CI 2.6%–25.4%) in Liberia, and 9.1% (95% CI 1.6%–19.1%) in Sierra Leone. This increase represents 713 (95% CI 58–1,528) additional deaths in Guinea, 155 (95% CI 23–297) in Liberia, and 223 (95% CI 29–504) in Sierra Leone (Table 3).

### Tuberculosis

Using a 50% reduction in treatment coverage for both drug susceptible and multidrug-resistant TB, we estimated that 3,463 (95% CI 2,808–4,349) persons would have died from TB in Guinea, 1,553 (95% CI 1,216–1,875) in Liberia, and 2,164 (95% CI 1,815–2,548) in Sierra Leone. The increase in TB deaths attributable to this reduction in TB treatment coverage was estimated to be 51.1% (95% CI 44.7%–70.5%) in Guinea, 59% (95% CI 47.9%–77.4%) in Liberia, and 61.4% (95% CI 49.2%–87.6%) in Sierra Leone. This increase represents 1,281 (95% CI 877–1,474) additional deaths in Guinea, 592 (95% CI 394–691) in Liberia, and 841 (95% CI 680–1,010) in Sierra Leone (Table 3).

### Variation in Treatment Coverage

We conducted a sensitivity analysis by varying the reduction of treatment coverage over a range of 10%–90% of the level before the Ebola outbreak for malaria, HIV/AIDS, and TB in Guinea, Liberia, and Sierra Leone. Because treatment coverage was varied, the additional deaths attributable to the Ebola outbreak in Guinea were estimated to vary from 1,425–8,336 for malaria, 146–1,237 for HIV/AIDS, and 277–2,317 for TB. In Liberia, additional deaths attributable to Ebola outbreak varied from 210–1,502 for malaria, 50–314 for HIV/AIDS, and 100–987 for TB. Additional death counts in Sierra Leone varied from 630–3,172 for malaria, 70–630 for HIV/AIDS, and 143–1,723 for TB.

**Figure 2:**
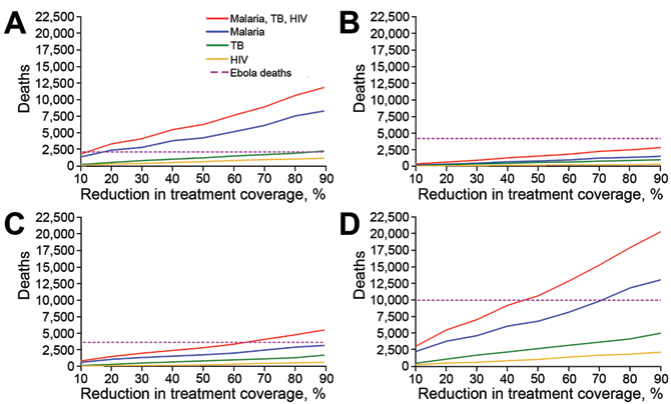
Sensitivity analysis of model outcomes to variation in treatment coverage during response to the 2014–2015 Ebola outbreak in West Africa. A) Guinea, B) Liberia, C) Sierra Leone, and D) all 3 countries. Treatment coverage of malaria, HIV/AIDS, and tuberculosis varied from 10% to 90% reduction compared with the coverage before the Ebola outbreak. Average additional attributable deaths from malaria, HIV/AIDS, and tuberculosis as well as total direct deaths from Ebola are shown. Estimates of additional attributable deaths were associated with considerable uncertainty and are not shown here.

We estimated that, for a reduction of treatment coverage of >15% in Guinea, the indirect deaths from malaria, HIV/AIDS, and TB associated with repercussions of Ebola exceeded the 2,170 cumulative death toll from Ebola reported in Guinea through March 8, 2015 (Figure 2, panel A) (*34*). In Liberia, the reported 4,162 direct deaths from Ebola (*34*) was likely greater than its indirect repercussions on malaria, HIV/AIDS, and TB (Figure 2, panel B). In Sierra Leone, a reduction in treatment coverage by >65% resulted in higher numbers of indirect deaths from malaria, HIV/AIDS, and TB than the reported 3,629 direct deaths from Ebola (Figure 2, panel C) (*34*). Overall, in the 3 countries studied in West Africa, a reduction in treatment coverage by 50% resulted in higher indirect deaths than direct deaths from Ebola (Figure 2, panel D).

### Multivariate Sensitivity Analysis

Our PRCC analysis demonstrated that the probabilities of dying from severe malaria with and without undergoing treatment, the probability of progressing from uncomplicated to severe malaria, the probability of dying from uncomplicated malaria without receiving treatment, and the probability of a child with a fever developing malaria have the greatest effects on additional malaria deaths attributable to Ebola for these 3 countries (Table 4). We found that the ART coverage before the Ebola outbreak and the probabilities of dying with and without undergoing ART for persons with a CD4 count <200 cells/mm^3^ were of most value in determining additional HIV/AIDS deaths attributable to Ebola (Table 4). Similarly, the treatment coverage for TB before the Ebola outbreak was found to be the principal parameter in determining deaths associated with TB (Table 4).

**Table 4:** Sensitivity of epidemiologic parameters to mortality rates from malaria, HIV/AIDS, and tuberculosis during response to the 2014–2015 Ebola outbreak in Guinea, Liberia, and Sierra Leone

Parameter	PRCC*		
	Guinea	Liberia	Sierra Leone
Malaria			
Death from severe malaria with treatment	−0.594	−0.751	−0.587
Death from severe malaria without treatment	0.822	0.626	0.411
Progressing from uncomplicated to severe malaria given no treatment	0.556	0.854	0.738
Death from uncomplicated malaria without treatment	−0.529	−0.785	−0.477
Fever cases attributable to malaria	0.470	0.701	0.415
Spontaneous recovery from uncomplicated malaria	0.173	0.305	0.289
Developing fever within 2 weeks at age <1	0.333	0.085	−0.018
Developing fever within 2 weeks at age 1–2	−0.145	−0.107	0.182
Developing fever within 2 weeks at age 2–3	−0.044	−0.009	−0.059
Developing fever within 2 weeks at age 3–4	0.139	0.287	−0.045
Developing fever within 2 weeks at age 4–5	−0.071	−0.315	−0.342
Treatment coverage for severe malaria	0.260	−0.093	0.444
Death from uncomplicated malaria with treatment	−0.396	−0.344	−0.411
Treatment coverage for malaria before Ebola outbreak for age <1	0.038	−0.104	0.211
Treatment coverage for malaria before Ebola outbreak for ages 1–2	0.139	−0.013	−0.163
Treatment coverage for malaria before Ebola outbreak for ages 2–3	0.071	0.143	0.313
Treatment coverage for malaria before Ebola outbreak for ages 3–4	−0.149	−0.030	−0.061
Treatment coverage for malaria before Ebola outbreak for ages 4–5	−0.455	−0.662	−0.239
HIV/AIDS			
Death while receiving ART and CD4 count <200 cells/mm^3^	−0.679	−0.734	−0.649
Treatment coverage for HIV/AIDS before Ebola outbreak	0.381	0.474	0.467
Death while receiving no treatment and CD4 count <200 cells/mm3	0.494	0.683	0.693
Population with CD4 count >500 cells/mm^3^	0.244	0.604	0.397
Population with CD4 count 350 to 499 cells/mm^3^	−0.168	−0.062	−0.165
Population with CD4 count 200 to 349 cells/mm^3^	−0.220	0.042	0.084
Death while receiving ART with CD4 count 350 to 499 cells/mm^3^	−0.204	−0.271	−0.313
Death while receiving ART with CD4 count 200 to 349 cells/mm^3^	−0.329	−0.451	−0.320
Death while receiving no treatment and CD4 count 350 to 499 cells/mm^3^	−0.295	−0.438	−0.367
Death while receiving no treatment and CD4 count 200 to 349 cells/mm^3^	0.107	0.116	−0.088
Tuberculosis			
Treatment coverage for tuberculosis before Ebola outbreak	0.916	0.894	0.946
Death while receiving treatment for MDR-TB	0.134	−0.243	0.148
Death while receiving no treatment for tuberculosis	0.255	−0.138	−0.002
Death while receiving treatment for DS-TB	0.030	−0.211	0.075
Clearing infection after treatment default	0.148	0.463	0.174
MDR–TB cases out of all new TB cases	0.154	−0.057	−0.214
Unsuccessful tuberculosis treatment (failure or default) for DS-TB	−0.037	−0.521	0.009
Treatment failure for DS-TB out of all unsuccessful treatment	−0.226	−0.479	0.177

*Partial rank correlation coefficients (PRCCs) were used to determine the association between the probability of mortality from the studied diseases and input parameters of the model. The PRCC of each parameter was statistically significant (p<0.001).

## Discussion

Our analysis estimates the extent to which the 2014–2015 Ebola outbreak in West Africa exacerbated the number of deaths from malaria, HIV/AIDS, and TB through reduced access to treatment. As unprecedentedly catastrophic as the Ebola outbreak has been, we estimated that these indirect repercussions of the Ebola outbreak may have been even greater than the deaths directly attributable to Ebola in Guinea, Sierra Leone, and Liberia. As little as a 15% reduction in treatment would lead to greater indirect than direct deaths from Ebola in Guinea, underscoring the importance of treating these endemic diseases and the fragility of the local healthcare system. A 65% reduction in treatment coverage would have been necessary to result in more deaths indirectly attributable than directly attributable to Ebola in Sierra Leone. In Liberia, although on average our estimates of indirectly attributable deaths due to Ebola were lower than directly attributable deaths, these estimates were subject to considerable uncertainty. For a 70% or more reduction in treatment, the upper range value of indirectly attributable deaths, 4,376, exceeded directly attributable Ebola deaths. At a more plausible reduction in treatment coverage of 50% for these 3 diseases, we estimated 6,269 (95% CI 2,564–12,407) additional deaths in Guinea, 1,535 (95% CI 522–2,878) in Liberia, and 2,819 (95% CI 844–4,844) in Sierra Leone.

The Ebola outbreak likely had the most detrimental effect on children with malaria, with an estimated 4,275 additional deaths among children <5 years of age in Guinea, 788 in Liberia, and 1,755 in Sierra Leone. Malaria is the most prevalent disease in West Africa and the primary cause of death among children. Although Ebola primarily affected young adults in West Africa (*35*), the indirect deaths were highest among young children who did not receive adequate treatment for malaria.

Our results are consistent with other studies that have indicated that the number of deaths caused by Ebola during his outbreak may have been surpassed by other viral diseases (*4*,*36*). For example, Walker et al. (*4*) found the number of additional deaths from malaria attributable to Ebola for a 50% reduction in healthcare capacity were estimated to be 2,700 (95% CI 1,400–5,200) in Guinea, 700 (95% CI 400–1,400) in Liberia, and 1,800 (95% CI 900–3,600) in Sierra Leone, which are consistent with our results.

For a 50% reduction in treatment coverage caused by healthcare deficiencies related to the Ebola outbreak, the percentage increase in malaria deaths was higher in Liberia (53.6%) compared with that of Guinea (48.0%) and Sierra Leone (50.0%). These differences are likely attributable to the higher pre-Ebola malaria treatment coverage in Liberia (51.1%); Guinea and Sierra Leone had 22.0% and 42.8% treatment coverage, respectively. The percentage increase in HIV/AIDS deaths attributable to Ebola was higher in Guinea (16.2%) than in Liberia (13.0%) and Sierra Leone (9.1%), consistent with higher pre-Ebola ART coverage in Guinea (51.3%), compared with Liberia (42.9%) and Sierra Leone (33.0%). For TB, the percentage increase in deaths attributable to Ebola was highest in Sierra Leone (61.4%) compared with Guinea (51.1%) and Liberia (59.0%), which corresponds with the high treatment coverage before the Ebola outbreak in Sierra Leone (66.5%) compared with Guinea (52.1%) and Liberia (54.8%).

Our analysis was conservative in several respects. Our base case estimates are conservative in the sense that some reports have cited much greater than 50% reduction in treatment accessibility due to the Ebola outbreak on healthcare systems in Guinea, Liberia, and Sierra Leone (*1*). We assumed no change in the transmission rate of malaria, HIV/AIDS, and TB during the course of the Ebola outbreak, due to a lack of data to estimate potential variation in disease transmission. This assumption may also be conservative because reduced treatment coverage may have elevated transmission; for example, viral loads in untreated HIV-positive persons would be expected to rise, concomitantly increasing risk for transmission (*3*,*4*). We also did not consider the effect of Ebola on reducing coverage of public health interventions such as bed nets and insecticide provision for malaria prevention, condoms and sexual health education to prevent HIV transmission, or Bacille Calmette-Guérin vaccination. In addition, we did not consider HIV/TB co-infection as a health state in our models. Although our study was conducted with the WHO recommendation for ART initiation at a CD4 count of 500 cells/mm^3^ in place during the timeframe of our study, it is possible that these guidelines were not yet being fully implemented in West Africa. Furthermore, our model considers only a short time horizon, limiting long-term measurements of the impact of the Ebola outbreak on HIV/AIDS, TB, and malaria deaths.

Because malaria may be highly seasonal in some West Africa countries, future studies should account for seasonality in malaria transmission to capture transient dynamics of annual malaria incidence and mortality rates (*29*). In West Africa, malaria transmission predominantly occurs during the rainy season, during April­­–December, which coincides with the peak of the Ebola outbreak in West Africa. This temporal overlap between the rainy season and the peak of the 2014–2015 Ebola outbreak exacerbated the indirect effect of Ebola on malaria in the 3 most affected countries (*4*).

Fear of nosocomial Ebola transmission may have deterred persons from seeking treatment for malaria, which has symptoms similar to Ebola, including fever, dizziness, and body aches (*1*). This problem was compounded by the unprecedented strain on the health systems of Guinea, Liberia, and Sierra Leon, which starkly limited provision of routine health services, such as childhood immunizations for vaccine-preventable diseases, obstetric care, and screening for sexually transmitted infections (*1*,*37*), as well as public health efforts against neglected tropical diseases (*38*). Thus, the burden of illness of the 2014–2015 Ebola outbreak will inevitably continue to include repercussions beyond deaths related to malaria, HIV/AIDS, and TB. These repercussions will continue long after our study period, caused by, for example, potential development of drug resistance and loss of vital healthcare workers. The societal burden from these diseases, which are beyond the scope of our analyses, extends beyond their direct health effect, yet is critical to perpetuating the vicious cycle of poverty and disease that leaves children unable to receive education and adults incapable of achieving their potential productivity and fully contributing to the development of their communities.

International donor organizations and governments, in combination with local community-based organizations, were instrumental to curtailing the Ebola outbreak in West Africa, without which more deaths directly attributable to Ebola, as well as further indirect devastation, would have occurred (*39*). Although public health officials rightfully focused efforts on curbing the Ebola outbreak, the long-term weakening of health systems related to the Ebola outbreak will require extensive investment directed at strengthening diffuse health systems for a plethora of diseases (*40*). Our analysis illustrates the need to invest resources in strengthening of health systems to mitigate vulnerability and reduce costs associated with health systems failing when stressed by acute events.

In conclusion, our results estimate that the 2014–2015 Ebola outbreak in West Africa has substantially impeded the fight against malaria, HIV/AIDS, and TB in the 3 countries most affected. As the Ebola outbreak wanes, it is essential for control strategies to include a comprehensive approach not only to stem the spread of Ebola, provide care for medical complications of recovered case-patients, and offer support for affected families but also to address the extensive repercussions of the outbreak that will continue long after Ebola elimination.

Technical Appendix Probabilities of treatment and outcomes dependent on CD4 counts of dying without antiretroviral treatment (ART), ART failure, and dying while receiving ART and description of population sizes used to calculate mortality rates from malaria, HIV/AIDS, and tuberculosis.
