# Supplementary material for: Effects of Response to 2014–2015 Ebola Outbreak on Deaths from Malaria, HIV/AIDS, and Tuberculosis, West Africa
Source: Emerg Infect Dis. 2016 Mar;22(3):433–41. doi: 10.3201/eid2203.150977 (PMC4766886; doi:10.3201/eid2203.150977)
Supplement: Technical Appendix — Probabilities of treatment and outcomes dependent on CD4 counts of dying without antiretroviral treatment (ART), ART failure, and dying while receiving ART and description of population sizes used to calculate mortality rates from malaria, HIV/AIDS, and tuberculosis. [file 15-0977-Techapp-s1.pdf]

Article DOI: http://dx.doi.org/10.3201/eid2203.150977

Effects of Response to the 2014–2015 Ebola Outbreak on Deaths from Malaria, HIV/AIDS, and Tuberculosis, West Africa

Technical Appendix

Technical Appendix Table 1. Parameter estimates for deaths from Malaria, HIV/AIDS, and tuberculosis in Guinea, Liberia, and Sierra Leone malaria models*

Variable		Uniform distribution range and β distribution values		Reference
Malaria-related parameter estimates: Guinea, Liberia, Sierra Leone				
Probability of death without treatment	Uncomplicated Malaria	0.005–0.02		(*1*)
	Severe Malaria	0.45–0.80		(*2*)
Probability of death while undergoing treatment	Uncomplicated Malaria	0.00024–0.00112		(*3*)
	Severe Malaria	0.05–0.2		(*4*)
Probability of progressing from uncomplicated to severe malaria given no treatment		0.03–0.13		(*2*,*5*)
Proportion of fever cases attributable to Malaria		0.01–0.11		(*5*,*6*)
Probability of spontaneous recovery from uncomplicated malaria		0.10–0.20		(*5*)
Probability of treatment for severe malaria		0.60–0.80		(*7*)
Guinea				
Age-specific probabilities of developing fever within 2 weeks (β distribution)		Cases	N	(*8*)
	<1 y	376	1,453	
	1–2 y	476	1,296	
	2–3 y	406	1,192	
	3–4 y	337	1,253	
	4–5 y	301	1,252	
Age-specific probabilities of receiving treatment for malaria before Ebola outbreak	<1 y	0.128–0.221		(*8*))
	1–2 y	0.194–0.334		
	2–3 y	0.159–0.260		
	3–4 y	0.198–0.309		
	4–5 y	0.163–0.271		
Liberia				
Age-specific probabilities of developing fever within 2 weeks (β distribution)		Cases	N	(*9*)
	<1 y	391	1,333	
	1–2 y	429	1,272	
	2–3 y	309	1,085	
	3–4 y	327	1,198	
	4–5 y	273	1,159	
Age-specific probabilities of receiving treatment for malaria before Ebola outbreak	<1 y	0.296–0.381		(*9*)
	1–2 y	0.461–0.603		
	2–3 y	0.393–0.538		
	3–4 y	0.449–0.618		
	4–5 y	0.521–0.624		
Sierra Leone				
Age-specific probabilities of developing fever within 2 weeks (β distribution)		Cases	N	(*10*)
	<1 y	576	2,406	
	1–2 y	706	2,169	
	2–3 y	570	2,011	
	3–4 y	493	2,237	
	4–5 y	406	1,991	
Age-specific probabilities of receiving treatment for malaria before Ebola outbreak	<1 y	0.301–0.395		(*10*)
	1–2 y	0.376–0.502		
	2–3 y	0.354–0.484		
	3–4 y	0.395–0.543		
	4–5 y	0.376–0.501		

Variable		Uniform distribution range and β distribution values	Reference
HIV/AIDS-related parameter estimates: Guinea, Liberia, and Sierra Leone			
CD4 count-specific probabilities of ART failure	CD4 499–350 cells/mm^3	0.03	(*11*)
	CD4 349–200 cells/mm^3	0.05	
	CD4 <200 cells/mm^3	0.1	
Population distributions of CD4 cells/mm^3 counts	CD4 >500 cells/mm^3	0.1833–0.44	(*12*–*14*)
	CD4 499–350 cells/mm^3	0.1430–0.22	
	CD4 349–200 cells/mm^3	0.1667–0.22	
	CD4 <200 cells/mm^3	0.12–0.42	
CD4 count-specific probabilities of death for those not treated	CD4 499–350 cells/mm^3	0.02–0.0926	(*11*,*15*–*17*)
	CD4 349–200 cells/mm^3	0.03–0.0926	
	CD4 <200 cells/mm^3	0.11254–0.25	
CD4 count-specific probabilities of death while receiving ART	CD4 499–350 cells/mm^3	0.00036–0.03915	(*15*,*18*,*19*)
	CD4 349–200 cells/mm^3	0.002397–0.0355	
	CD4 <200 cells/mm^3	0.037731–0.160971	
Guinea			
Probability of receiving ART	Prior to Ebola outbreak	0.43–0.58	(*20*,*21*)
Liberia			
Probability of receiving ART	Prior to Ebola outbreak	0.38–0.48	(*20*,*21*)
Sierra Leone			
Probability of receiving ART	Prior to Ebola outbreak	0.24–0.42	(*20*,*21*)
Tuberculosis-related parameter estimates: Guinea, Liberia, and Sierra Leone			
Probability of death without treatment		0.20–0.40	(*22*,*23*)
Probability of death while undergoing treatment	DS-TB	0.02–0.08	(*24*)
	MDR-TB	0.09–0.13	(*25*)
Probability of Treatment Failure	DS-TB	0.001–0.004	(*26*)
	MDR-TB	0.05–0.11	
Probability of defaulting on treatment	DS-TB	0.04–0.15	(*27*)
Probability of progressing to MDR-TB	Experienced treatment failure	0.173	(*26*)
	Defaulted on treatment	0.023	
Probability of clearing infection after treatment default		0.20–0.30	(*23*)
Guinea			
Proportion of all new TB cases that are MDR-TB		0.001–0.016	(*28*)
Probability of receiving treatment for TB	Prior to Ebola outbreak	0.48–0.61	(*20*,*21*)
Liberia			
Proportion of all new TB cases that are MDR-TB		0.001–0.053	(*28*)
Probability of receiving treatment for TB	Prior to Ebola outbreak	0.51–0.64	(*20*,*21*)
Sierra Leone			
Proportion of all new TB cases that are MDR-TB		0–0.047	(*28*)
Probability of receiving treatment for TB	Prior to Ebola outbreak	0.50–0.83	(*20*,*21*)

*ART, antiretroviral treatment; DS-TB, drug-susceptible tuberculosis; MDR-TB, multidrug-resistant tuberculosis.

Technical Appendix Table 2. Population sizes used in models to calculate mortality rates from Malaria, HIV/AIDS, and Tuberculosis, West Africa

Category		Guinea		Liberia		Sierra Leone	
		Population	Ref	Population	Ref	Population	Ref
Population at risk for Malaria (age <5)*	Mean	1,900,000	(29)	700,000	(29)	900,000	(*29*)
HIV-infected population (age 15–49)	Mean	97,000	(30)	22,000	(31,32)	48,000	(*31*,*33*)
	95%CI	81,000–120,000		18,000–26,000		36,000–60,000	
Population with active TB, without HIV/AIDS (all ages)	Mean	21,755	(34)	16,669	(35)	23,846	(*36*)
	95%CI	11,858–34,504		8,895–26,798		11,922–39,794	

*For the malaria model, we used the national population size of persons<5 y old. Ref, reference.

References

1. Rafael ME, Taylor T, Magill A, Lim Y-W, Girosi F, Allan R. Reducing the burden of childhood malaria in Africa: the role of improved. Nature. 2006;444(Suppl 1):39–48. PubMed http://dx.doi.org/10.1038/nature05445

2. Lubell Y, Staedke SG, Greenwood BM, Kamya MR, Molyneux M, Newton PN, et al. Likely health outcomes for untreated acute febrile illness in the tropics in decision and economic models; a Delphi survey. [Internet]. PLoS ONE. 2011;6. http://dx.doi.org/10.1371/journal.pone.0017439

3. Thwing J, Eisele TP, Steketee RW. Protective efficacy of malaria case management and intermittent preventive treatment for preventing malaria mortality in children: a systematic review for the Lives Saved Tool. BMC Public Health. BioMed Central Ltd; 2011;11 Suppl 3(Suppl 3):S14.

4. von Seidlein L, Olaosebikan R, Hendriksen ICE, Lee SJ, Adedoyin OT, Agbenyega T, et al. Predicting the clinical outcome of severe falciparum malaria in african children: findings from a large randomized trial. Clin Infect Dis. 2012;54:1080–90. PubMed http://dx.doi.org/10.1093/cid/cis034

5. Mori AT, Ngalesoni F, Norheim OF, Robberstad B. Cost-effectiveness of dihydroartemisinin-piperaquine compared with artemether-lumefantrine for treating uncomplicated malaria in children at a district hospital in Tanzania. Malar J. 2014;13:363. PubMed http://dx.doi.org/10.1186/1475-2875-13-363

6. Smith T, Schellenberg JA, Hayes R. Attributable fraction estimates and case definitions for malaria in endemic areas. Stat Med. 1994;13:2345–58. PubMed http://dx.doi.org/10.1002/sim.4780132206

7. de Savigny D, Mayombana C, Mwageni E, Masanja H, Minhaj A, Mkilindi Y, et al. Care-seeking patterns for fatal malaria in Tanzania. Malar J. 2004;3:27. PubMed http://dx.doi.org/10.1186/1475-2875-3-27

8. Institut National de la Statistique de Guinée, Measure DHS ICF International. Enquête Démographique et de Santé et à Indicateurs Multiples (EDS-MICS 2012) - Guinée 2012. Conakry, Guinée; 2013 [cited 2015 May 1]. http://dhsprogram.com/pubs/pdf/FR280/FR280.pdf

9. Liberia Institute of Statistics and Geo-Information Services (LISGIS). Liberia Demographic and Health Survey 2013. 2014 [cited 2015 May 1]. https://dhsprogram.com/pubs/pdf/FR291/FR291.pdf

10. Statistics Sierra Leone, Ministry of Health and Sanitation, ICF International. Sierra Leone demographic and health survey 2013. Freetown, Sierra Leone, and Rockville (Maryland, USA); 2014. p. 36. http://microdata.worldbank.org/index.php/catalog/2167

11. Stover J, Bollinger L, Avila C. Estimating the effects and costs of changing guidelines for ART eligibility. Geneva: World Health Organization. 2009;(Sep):1–18 [cited 2015 Jun 2]. http://www.who.int/hiv/topics/treatment/costing_stover.pdf

12. Akinbami A, Dosunmu A, Adediran A, Ajibola S, Oshinaike O, Wright K, et al. CD4 count pattern and demographic distribution of treatment-naive hiv patients in Lagos, Nigeria. AIDS Res Treat. 2012;2012 [cited 2015 May 1]. http://www.hindawi.com/journals/art/2012/352753/

13. National AIDS and STI Control Programme. Kenya AIDS Indicator Survey 2007. Kenya: Kenya Ministry of Health; 2008.

14. Williams BG, Korenromp EL, Gouws E, Schmid GP, Auvert B, Dye C. HIV infection, antiretroviral therapy, and CD4+ cell count distributions in African populations. J Infect Dis. 2006;194:1450–8. PubMed http://dx.doi.org/10.1086/508206

15. Badri M, Cleary S, Maartens G, Pitt J, Bekker LG, Orrell C, et al. When to initiate highly active antiretroviral therapy in sub-Saharan Africa? A South African cost-effectiveness study. Antivir Ther. 2006;11:63–72. PubMed

16. Hamers RL, Sawyer W, Tuohy M, Stevens WS, de Wit TFR, Hill AM. Cost-effectiveness of laboratory monitoring for management of HIV treatment in sub-Saharan Africa. AIDS. 2012;26:1663–72. PubMed http://dx.doi.org/10.1097/QAD.0b013e3283560678

17. Lewden C, Gabillard D, Minga A, Ekouévi DK, Avit D, Konate I, et al. CD4-specific mortality rates among HIV-infected adults with high CD4 counts and no antiretroviral treatment in West Africa. J Acquir Immune Defic Syndr. 2012;59:213–9. PubMed http://dx.doi.org/10.1097/QAI.0b013e31823b837e

18. Lawn SD, Little F, Bekker L-G, Kaplan R, Campbel E, Orrell C, et al. Changing mortality risk associated with CD4 cell response to antiretroviral therapy in South Africa. AIDS. 2009;23:335–42. PubMed

19. Mills EJ, Bakanda C, Birungi J, Mwesigwa R, Chan K, Ford N, et al. Mortality by baseline CD4 cell count among HIV patients initiating antiretroviral therapy: evidence from a large cohort in Uganda. AIDS. 2011;25:851–5. PubMed http://dx.doi.org/10.1097/QAD.0b013e32834564e9

20. World Health Organization Regional Office for Africa. African Regional Health Report 2014: Health Statistics. Brazzaville, Republic of Congo: World Health Organization; 2104.

21. World Health Organization. Global Health Observatory Data Repository: Statistics Summary (2002-present). 2013 [cited 2015 May 1]. http://apps.who.int/gho/data/node.country

22. Dye C, Williams BG. Criteria for the control of drug-resistant tuberculosis. Proc Natl Acad Sci U S A. 2000;97:8180–5. PubMed http://dx.doi.org/10.1073/pnas.140102797

23. Law S, Benedetti A, Oxlade O, Schwartzman K, Menzies D. Comparing cost-effectiveness of standardised tuberculosis treatments given varying drug resistance. Eur Respir J. 2014;43:566–81. PubMed http://dx.doi.org/10.1183/09031936.00005613

24. World Health Organization. Global Tuberculosis Report 2014. Geneva, Switzerland: World Health Organization; 2014.

25. Johnston JC, Shahidi NC, Sadatsafavi M, Fitzgerald JM. Treatment outcomes of multidrug-resistant tuberculosis: A systematic review and meta-analysis. [Internet]. PLoS ONE. 2009;4: http://dx.doi.org/10.1371/journal.pone.0006914. PubMed

26. Menzies D, Benedetti A, Paydar A, Martin I, Royce S, Pai M, et al. Effect of duration and intermittency of rifampin on tuberculosis treatment outcomes: A systematic review and meta-analysis. PLoS Med. 2009;6:1–18. PubMed http://dx.doi.org/10.1371/journal.pmed.1000146

27. World Health Organization. World Malaria Report 2014. Geneva, Switzerland: Global Malaria Programme, World Health Organization; 2014 Feb p. 1–227.

28. World Health Organization. Tuberculosis Profile [Internet]. 2013 [cited 2015 May 1]. http://www.who.int/tb/country/data/profiles/en/

29. United Nations Development Programme. Population aged under 5 (millions) [Internet]. United Nations Development Programme Human Development Reports. 2013 [cited 2015 May 1]. http://hdr.undp.org/en/content/population-aged-under-5-millions

30. UNAIDS. Developing estimates of HIV prevalence and the number of people living with HIV. 2014 [cited 2015 May 1]. http://www.unaids.org/sites/default/files/media_asset/2014_subnationalestimatessurvey_Guinea_en.pdf

31. Population Reference Bureau. Population of Women Ages 15–49 in Millions [Internet]. Population Reference Bureau. 2013 [cited 2015 May 1]. http://www.prb.org/DataFinder/Topic/Rankings.aspx?ind=18

32. Joint United Nations Programme on HIV/AIDS. Liberia: HIV and AIDS estimates. UNAIDS; 2013.

33. UNAIDS. Sierra Leone: HIV and AIDS estimates. 2013 [cited 2015 May 1]. http://www.unaids.org/sites/default/files/epidocuments/SLE.pdf

34. World Health Organization. Guinea: Tuberculosis Profile. 2013 [cited 2015 May 1]. https://extranet.who.int/sree/Reports?op=Replet&name=/WHO_HQ_Reports/G2/PROD/EXT/TBCountryProfile&ISO2=GN&outtype=html

35. World Health Organization. Liberia: Tuberculosis Profile. 2013 [cited 2015 May 1]. https://extranet.who.int/sree/Reports?op=Replet&name=/WHO_HQ_Reports/G2/PROD/EXT/TBCountryProfile&ISO2=LR&outtype=html

36. World Health Organization. Sierra Leone: Tuberculosis Profile. 2013 [cited 2015 May 1]. https://extranet.who.int/sree/Reports?op=Replet&name=/WHO_HQ_Reports/G2/PROD/EXT/TBCountryProfile&ISO2=SL&outtype=html
